# YULINK deficiency promotes cell death under glucose restriction in HCC cells in association with GLUT1-mediated glycolysis

**DOI:** 10.1186/s10020-025-01347-7

**Published:** 2025-09-26

**Authors:** Yi-Chia Wu, Tsai-Hsien Hung, Wei-Ting Thomas Wang, Ming-Wei Kuo, Yuh-Jin Liang, Yur-Ren Kuo, Ming-Feng Hou, Chung-Sheng Lai, Alice L. Yu, John Yu

**Affiliations:** 1 Institute of Stem Cell and Translational Cancer Research, Chang Gung Memorial Hospital at Linko, Taoyuan, Taiwan; 2 Regenerative Medicine and Cell Therapy Research Center, Kaohsiung Medical University, Kaohsiung, Taiwan; 3 Division of Plastic Surgery, Department of Surgery, Kaohsiung Medical University Hospital, Kaohsiung Medical University, Kaohsiung, Taiwan; 4 Division of Breast Oncology and Surgery, Department of Surgery, Kaohsiung Medical University Hospital, Kaohsiung Medical University, Kaohsiung, Taiwan; 5 School of Medicine, Kaohsiung Medical University, Kaohsiung, Taiwan; 6 Chang Gung University, #15, Wenhua 1st Rd, Guishan Dist, Taoyuan, 333 Taiwan; 7 Department of Pediatrics, University of California in San Diego, San Diego, CA 92103 USA

**Keywords:** YULINK, Hepatocellular carcinoma, Glucose transporter isoform 1, Glucose restriction

## Abstract

**Background:**

Through evolutionary genomics analysis, we identified Yulink (MIOS, Entrez Gene: 54,468), a highly conserved gene encoding an 875 amino acid protein with diverse functions in humans. Given the importance of accelerated glycolysis in hepatocellular carcinoma (HCC), we explored the expression and function of Yulink in HCC cells and analyzed clinicopathological data to unveil its impact on patient survival.

**Methods:**

Clinicopathological data from 184 patients with resectable HCC were mined to establish a correlation between Yulink expression and patient survival. We employed reverse transcription quantitative polymerase chain reaction (RT-qPCR) to assess Yulink expression in the tumor tissues. Various assays, including Western blotting, migration, MTT, cell cycle, immunofluorescence, oxidative stress, tumorigenesis, glucose uptake, glycolytic function, proximity ligation, and immunoprecipitation, were conducted on Huh7 cells to identify the regulatory mechanisms under glucose restriction.

**Results:**

Comparative evolutionary genomics analysis revealed that patients with high Yulink expression had significantly shorter relapse-free survival (RFS) and overall survival (OS) (*P* < 0.0001 and = 0.0015, respectively). Multivariable Cox regression analysis identified Yulink expression as an independent unfavorable predictor of RFS (HR, 2.63; 95% CI, 1.58–4.38; *P* < 0.001) in HCC. Furthermore, Yulink expression positively correlated with Huh7 migration and survival, especially in response to glucose restriction. Yulink deficiency enhanced glucose restriction-induced cell death, likely due to increased reactive oxygen species (ROS) and DNA damage, with a failure of ATM-CHK2 activation. Huh7 xenografts with Yulink suppression exhibited delayed tumorigenesis in immunocompromised nude mice. Importantly, proximity Ligation assays and immunoprecipitation demonstrated that Yulink colocalized and interacted with glucose transporter 1 (GLUT1). Knockdown of Yulink not only suppressed GLUT1 expression, but also disrupted GLUT1 translocation from the cytosol to the cell membrane, resulting in downregulated glucose uptake and glycolysis.

**Conclusions:**

Our results underscore the protective role of Yulink in HCC survival under glucose restriction and its pivotal function in glucose metabolism, suggesting a mechanistic link between lower Yulink expression and higher survival in patients with HCC.

**Supplementary Information:**

The online version contains supplementary material available at 10.1186/s10020-025-01347-7.

## Background

Hepatocellular carcinoma (HCC) is one of the leading causes of death worldwide, and its incidence is increasing, especially in the USA and most European countries. Most HCC cases are diagnosed in a late clinical state (Alves et al. 2019). The diagnosis and management of HCC has progressed enormously in recent decades. Surgical resection or liver transplantation, percutaneous radiofrequency ablation, and systemic chemotherapy are the most common therapeutic strategies for HCC (Vogel et al. 2022). However, owing to poor treatment outcomes, it is still the third deadliest cancer, with over 780,000 deaths per year worldwide (Kusnik et al. 2021). Therefore, there is an urgent need to develop novel therapeutic strategies against HCC.

Previously, we identified an evolutionarily conserved gene named YULINK (MIOS, Entrez Gene: 54,468) using comparative evolutionary genomics analysis (Kuo et al. 2021). The gene encodes an 876 amino acid protein (96 kDa), containing an N-terminus with WD40 repeats, and a highly conserved segment near the C terminus with a missing oocyte (mio) domain. Recently, we demonstrated that YULINK plays a critical role in glucose metabolism, particularly in functional glycolysis, which is essential for cancer cell proliferation and migration (Wu et al. 2023). Additionally, YULINK has also been reported to interact with other proteins, including WDR24, WDR59, Seh1L and Sec13, to form a complex which serves as a regulator of mTOR signaling (Valenstein et al. 2022). Because mTOR plays an important role in nutrient sensing and cell metabolism (Goul et al. 2023; Chen et al. 2021; Wang et al. 2021), it may act as a regulator of energy homeostasis, particularly metabolic regulation in cancer cells.

Glucose restriction commonly occurs in the microenvironment of solid tumors because of enhanced glycolytic flux (Antoun et al. 2020). Cancer cells adapt their metabolic functions to restricted nutrition, promote tumor progression, and enhance tumor survival pathways (Martínez-Reyes and Chandel 2021). HCC cells are metabolically different from primary hepatocytes in that they exhibit high glucose uptake and accelerated glycolysis, known as the Warburg effect. Glucose transporter isoform 1 (GLUT1) is the major glucose transporter in HCC cells, whereas the predominant glucose transporter in normal hepatocytes is GLUT2 (Lei et al. 2020). Kaplan–Meier survival curves showed that HCC patients with high GLUT1 expression were associated with lower overall survival than those with low expression levels of GLUT1. These facts strongly suggest that YULINK, a metabolic regulator, may play a crucial role in cancerous glucose metabolism and HCC development.

In the current study, we examined the role of YULINK in HCC clinicopathology, especially in glucose metabolism and metabolic stress-triggered DNA damage. Here, our clinical results show that YULINK expression is negatively correlated with the overall and relapse-free survival of patients with HCC. YULINK knockdown in Huh7 cells resulted in decreased cell proliferation, migration, glucose uptake, and glycolytic function, which might be related to the interaction between YULINK and GLUT1.

## Methods

### Clinical specimens

RNA samples and clinical and pathological data were collected from 184 individuals diagnosed with stage I to IV hepatocellular carcinoma (HCC), and staging was determined according to the American Joint Committee on Cancer guidelines (Chun et al. 2018). These samples were sourced from the Tissue Bank of Linkou Chang Gung Memorial Hospital in Taoyuan, Taiwan as well as from the Taiwan Liver Cancer Network. All 184 samples analyzed were obtained from treatment-naïve patients and represented primary tumors. Prior to tissue deposition, all participants provided written informed consent. All research procedures were conducted in compliance with relevant guidelines and regulations and were approved by the Institutional Review Board of Chang Gung Medical Foundation (IRB number: 201304758B0), Review Board of Tri-Service General Hospital, and Biobank Ethics Committee of the National Health Research Institutes. RNA sequencing data of TCGA samples were downloaded from THE HUMAN PROTEIN ATLAS website.

### Quantitative reverse-transcription real-time polymerase chain reaction

Total RNA (1 μg) was converted to cDNA using a cDNA reverse transcription kit (Applied Biosystems, CA, USA), according to the manufacturer’s instructions. Then The expression level of YULINK was determined using SYBR Green Real-Time PCR Master Mix on an Applied Biosystems 7500 Fast Real-Time PCR System with the reaction conditions of pre-incubation at 95 °C for 10 s to activate HotStart Taq DNA polymerase, followed by 40 cycles of 15 s at 95 °C, and 1 min at 60 °C. Ten nanograms of cDNA from the tumors were used. Nuclease-free water was used to replace the cDNA in each run as a negative control for PCR amplification. Glyceraldehyde-3-phosphate dehydrogenase (GAPDH) and ubiquitin C (UBC) served as endogenous controls. The fluorescent signals were analyzed using 7500 software v2.06 (Applied Biosystems). After subtracting the geometric mean of the two reference control genes, the results of the qRT-PCR analyses are shown as − ΔCT. The prognostic performance of the genes was calculated using the receiver operating characteristic (ROC) curve and area under the ROC curve. The Youden index (sensitivity + specificity − 1) was used to determine the optimal cut-off value for high versus low YULINK expression levels (Kuo et al. 2017). Based on this approach, the specific cutoff value of − 6.1685 was used to stratify patients. These tests were two-sided, and P-values < 0.05 were considered statistically significant. Statistical analyses were performed using Prism 5.0 (GraphPad Software, CA, U.S.A.).

### Primer sequences for Q-PCR

Human YULINK:

- F: AATCAATTGTAAAGTCATCGTTGG
- R: GCTTATCCAACCCACTCCAAT

Human GAPDH:

- F: GTCTCCTCTGACTTCAACAGCG
- R: ACCACCCTGTTGCTGTAGCCAA

Human UBC:

- F: ATTTGGGTCGCGGTTCTTG
- R: TGCCTTGACATTCTCGATGGT

### Cell line and culture

The Huh7 cell line was used in vitro and in vivo since it is one of the most widely used and well-characterized human HCC cell lines, possessing typical features such as high tumorigenic potential. Huh7 cells were maintained in high glucose (25 mM) Dulbecco's modified Eagle's medium (DMEM) supplemented with 10% fetal bovine serum (FBS) (Gibco 26,140–079, MA, U.S.A.), 1% antibiotic–antimycotic (Gibco 15,240–062) and 1% L-glutamine (Gibco 25,030,081) in a humidified incubator at 37℃ with 5% CO_2_. Confluent cells were separated by incubation with 0.05% trypsin–EDTA (Gibco 15,400–054). Furthermore, cells were cultured in glucose-restricted conditions with 1 mM glucose in DMEM (Gibco 11,966–025) supplemented with 10% FBS and 1% antibiotic–antimycotic (15,140,122, Thermo Fisher Scientific, MA, U.S.A.).

### YULINK knockdown

Scramble control and YULINK shRNA were packaged into pGIPz lentiviral shRNAmir vectors (Clone ID: V3LHS_374795 and gene access no. NM_019005, respectively), along with group-specific antigens, reverse transcriptase (gag-pol), and VSV-G-expressing plasmids (Open Biosystems/Thermo Scientific, AL, U.S.A.). Phoenix packaging cells were used with the CellPhect transfection kit (Amersham Biosciences, Buckinghamshire, U.K.). Viral supernatant was collected 48 h after transfection, filtered through 0.45-μm filters, and added to target cells for 72 h along with 8 μg/ml polybrene. YULINK knockdown cells were selected with 4 μg/ml puromycin to generate stable lines and maintained in media containing antibiotics.

### YULINK overexpression

The coding region of human YULINK was cloned into a lentiviral pCDH-CMV-MCS-EF1α-copGFP vector (System Biosciences, CA, U.S.A.). YULINK overexpression (OE) cells were generated by lentivirus applied to Huh7 cells with 8 μg/ml of polybrene (Sigma-Aldrich, MO, U.S.A).

### Transwell migration assay

Transwell Permeable Supports (3422, CORNING, NY, USA) were used to examine cell migration. Huh7 cells (1 × 10^4^ cells), with or without YULINK overexpression/knockdown, were plated on the upper layer of the cell culture insert coated with a permeable membrane for attachment. The coating was inserted into the culture well with normal or glucose restriction for 24 h. The cells were washed with PBS and fixed with 4% paraformaldehyde. The fixed cells were washed and stained with crystal violet. Cells that had not migrated were wiped off using cotton swabs. The migrated cells were photographed and recorded using a microscope.

### Cell cycle analysis

Cells, including floating cells, were collected and fixed with 70% ethanol to increase the permeability of propidium iodide staining. RNase A was used to eliminate any possible RNA interference. The SubG1 population and whole cell cycle distribution were analyzed using an LSR II Flow Cytometer (BD Biosciences) and FlowJo software (FlowJo LLC, OR, U.S.A.).

### Cell proliferation and viability assay

Cell proliferation and viability were examined using the 3-(4,5-dimethylthiazol-2-yl)−2,5-diphenyltetrazolium bromide (MTT) assay. The cells were washed and cultured in fresh medium containing 10 μg/ml MTT for 2 h in a 37℃, 5% CO_2_ incubator. Formazan crystals produced by living cells were dissolved in dimethyl sulfoxide (DMSO) and quantified by measuring the optical density at a wavelength of 570 nm.

### Immunofluorescence staining

The cells were grown on glass coverslips and fixed with 3% buffered paraformaldehyde for immunostaining. After staining, the cells were analyzed using a Zeiss Axioplan 2 Upright Fluorescent Microscope and Volocity 3.6.1 (Improvision, West Midlands, U.K.) software. The primary antibody was γ-H2AX pSer 139 (ab2893, Abcam, Cambridge, U.K.) and the secondary antibody was Alexa Fluor 594 goat anti-rabbit IgG (H + L) (A11012, Thermo Fisher Scientific).

### Oxidative stress determination

Oxidative stress was examined using the oxidative stress indicator CM-H2DCFDA (C6827, Thermo Fisher Scientific). Cells were detached using 0.25% trypsin–EDTA and collected in pre-warmed phosphate buffered saline (PBS). Approximately 2 × 10^6^ cells were incubated in PBS with freshly prepared 10 μM CM-H2DCFDA at 37℃ for 1 h. Cells were then washed once with PBS and analyzed using an LSR II Flow Cytometer. Cellular ROS levels were determined by measuring the emission of wavelengths around 517 nm, which were excited at 488 nm.

### Western blot

Protein lysates were prepared using RIPA lysis buffer containing 1 M phenylmethylsulfonyl fluoride (PMSF), PhosSTOP Phosphatase Inhibitor Cocktail (PHOSS-RO, Sigma-Aldrich), and β-mercaptoethanol. Lysates were then fractionated by sodium dodecyl sulfate–polyacrylamide gel electrophoresis (SDS-PAGE) and transferred to PVDF membranes. After blocking, the membranes were blotted with antibodies and detected using the Western Lightning® Plus-ECL detection system (NEL104001EA, PerkinElmer, MA, U.S.A.). The primary antibodies used were anti-YULINK/MIOS (ab202274, Abcam, 135,575, Cell Signaling, MA, U.S.A.), anti-PI3K p85 pTyr 458/p55 pTyr 199 (4228, Cell Signaling), anti-PI3K p110α (4249, Cell Signaling), anti-AKT pSer 473 (4060, Cell Signaling), anti-AKT pThr 308 (13,038, Cell Signaling), anti-AKT (4685, Cell Signaling), anti-CHK2 pThr 68 (2661, Cell Signaling), anti-CHK2 (3440, Cell Signaling), anti-ATM pSer 1981 (5883, Cell Signaling), anti-γ-H2AX (ab2893, Abcam), and anti-GAPDH (MAB374, Millipore, MA, U.S.A.). The secondary antibodies used were anti-rabbit IgG HRP-linked antibody (7074, Cell Signaling) and anti-mouse IgG HRP-linked antibody (7076, Cell Signaling). The blot intensities were quantified using ImageJ software.

### Animals and tumorigenesis

This animal study performed in this manuscript was approved by the Institutional Animal Care and Use Committee of Kaohsiung Medical University (IACUC-106257). Fifteen adult male nude mice (BALB/c AnN. Cg-Foxnlnu/CrINarl) were provided by the National Laboratory Animal Breeding and Research Center (Taipei, Taiwan) and housed under standard conditions with ad libitum for subsequent experiments.

For tumorigenesis analysis, mice were randomly divided into two groups. A total of 1 × 10^6^ Huh7 cells with YULINK knockdown (n = 8) or scramble control (n = 7) were mixed with Matrigel to a final volume of 50 μl and subcutaneously injected into the dorsal region of nude mice. Tumor formation was monitored for up to eight weeks. After sacrifice, tumor volume was calculated using the formula: (length × width^2^)/2.

### 2-NBDG glucose uptake analysis

Cells were plated into 24-well culture plates at 2.5 × 10^4^ cells/well and maintained overnight to allow attachment. On the day of analysis, the regular culture medium (10% FBS) was replaced with 0.5% FBS culture medium for 1-h of starvation. Afterwards, cells were treated with “glucose uptake mix” containing fluorescent 2-deoxy-2-[(7-nitro-2,1,3-benzoxadiazol-4-yl) amino]-D-glucose (2-NBDG) (Glucose Uptake Assay Kit, K682-50, BioVision, CA, U.S.A.) for 15 min. The treated cells were collected and washed with ice-cold analysis buffer. The level of glucose uptake was determined using an LSR II Flow Cytometer (BD Biosciences, NJ, U.S.A.) with a 488 nm wavelength excitation filter. Because 2-NBDG cannot be utilized in glycolysis, the amount of FITC fluorescence, as measured by the intracellular accumulation of 2-NBDG, indicates the level of glucose uptake.

### Glucose uptake assay

Since the green fluorescence of GFP was similar to that of 2-NBDG, we used the Screen Quest™ Fluorimetric Glucose Uptake kit (AAT Bioquest, CA, U.S.A.) with red light to examine YULINK overexpression-related glucose uptake. Huh7 cells with or without YULINK overexpression were plated in 96-well culture plates at 8 × 10^4^ cells/well overnight to allow attachment. Afterwards, cells were starved for 6-h starvation period and treated with 2-DG for 30 min, followed by incubation with 2-DG Uptake Assay working solution. In this assay, 2-DG is taken up by cells and metabolized to non-metabolizable 2-DG-6-phosphate (2-DG6P). The accumulated 2-DG6P was subsequently oxidized by an enzymatic reaction to produce NADPH, which was then detected via fluorescence at 540/590 nm.

### Glycolytic function analysis

The extracellular acidification rate in either scramble control or YULINK knockdown cells was examined using a Seahorse Bioscience XF24 Extracellular Flux Analyzer (Seahorse Bioscience, MA, U.S.A.) and a Seahorse XF Glycolysis Stress Test Kit (103,020–100, Seahorse Bioscience) according to the manufacturer’s instructions. In general, 3 × 10^4^ cells/well were prepared in XF24 plates the day prior to the assay to allow for cell attachment. The cells were then refreshed with the analytical solution and examined with the XF24 analyzer.

### Proximity ligation assay

Huh7 cells were grown on slides and cultured in DMEM containing 25 or 1 mM glucose. The cells were fixed, permeabilized, and examined using the Duolink® In Situ Red Starter Kit (DUO92101, Sigma-Aldrich, MO, U.S.A.) according to the manufacturer’s instructions. Protein–protein interactions were observed using Leica TCS SP8 confocal microsystems (TCS SP8, Leica, Hessen, Germany).

### Immunoprecipitation analysis

Immunoprecipitation analysis was performed using the Pierce Crosslink Magnetic IP/Co-IP Kit (88,805, Thermo Fisher Scientific) following manual guidelines. Magnetic beads were washed with 1 × Modified Coupling Buffer before incubation with the antibody. The bound antibodies were then crosslinked to the beads with DSS for 30 min. The beads were washed with Elution Buffer followed by two additional washes with IP Lysis/Wash Buffer. Afterwards, pre-prepared Huh7 cell lysates were incubated with antibody-cross-linked beads for 1–2 h at room temperature before bound antigen elution.

### Membrane and cytosolic proteins extraction

Huh7 cells with or without YULINK knockdown were cultured in 25 mM or 1 mM glucose in DMEM for 24 h. Cells were collected for protein extraction. Membrane and cytosolic protein extractions were performed according to the manufacturer’s instructions (Mem-PER Plus Membrane Protein Extraction Kit, 89,842, Thermo Fisher Scientific).

### Statistical analysis

IBM SPSS statistics 25 (one-way ANOVA followed by post-hoc analysis with Bonferroni correction; SPSS Inc., IL, U.S.A.) was used for statistical analysis. All the reported results were derived from at least three replicates. The Youden index (sensitivity + specificity − 1) was used to determine the optimal cutoff value for high versus low gene expression levels. Survival curves were plotted using the Kaplan–Meier method, with the log-rank test applied for comparison. The Cox proportional-hazards regression model was used to evaluate the independent prognostic factors. Variable selection for the multivariate Cox regression models was conducted using a likelihood ratio-based stepwise forward selection method, incorporating variables with *P*-values < 0.1 in the univariate analysis. Multicollinearity among variables was assessed by calculating variance inflation factors (VIFs); all VIFs were < 2, indicating no significant multicollinearity. Values are presented as the mean ± S.D. from at least three independent experiments. Statistical significance was set at *P* < 0.05.

## Results

### YULINK expression in patients with HCC

To evaluate the clinical relevance of YULINK expression, we examined the clinical characteristics and demographic information of 184 patients with HCC (Table 1). The mean age was 56.0 ± 13.9 year with 82.6% being male. Among the patients, 40.6% had disease recurrence and 96.2% had chronic hepatitis infection, with the majority being infected by HBV. Liver cirrhosis was observed in 42.9% of the patients. Tumor sizes ranged from 0.8 to 18 cm with a mean size of 6.3 ± 4.2 cm, but less than 5 cm in 53.3% of cases. Furthermore, 51.1% had histological grade 1–2 tumors and 51.6% showed evidence of vascular invasion in their tumors. According to the tumor-node-metastasis (TNM) (Chun et al. 2018), 65.8% of the patients had tumors with TNM stages I and II. Tumor metastasis was observed in 15.7% of patients at diagnosis. The median follow-up time was 29.91 months.

**Table 1:** Clinical and pathological characteristics of 184 HCC patients

Characteristics	N (%)	Characteristics	N (%)
**Age**	56.0 ± 13.9 (19–83)	**Vascular invasion**	
**Gender**		Absent	89 (48.4%)
Male	152 (82.6%)	Present	95 (51.6%)
Female	32 (17.4%)	**Tumor Number**^**c**^	
**Drinking history**		Solitary	126 (88.3%)
Yes	56 (30.4%)	multiple	35 (21.7%)
No	128 (69.6%)	**Cirrhosis**	
**Virus infection**		No	105 (57.1%)
None	7 (3.8%)	Yes	79 (42.9%)
HBV	136 (73.9%)	**Metastasis**	
HCV	33 (17.9%)	No	157 (85.3%)
HBV + HCV	8 (4.4%)	Yes	27 (15.7%)
**Tumor size (cm)**	6.3 ± 4.2 (0.8–18)	**Relaspe**^**d**^	
≤ 5 cm	98 (53.3%)	No	73 (40.6%)
> 5 cm	86 (46.7%)	Yes	107 (59.4%)
**Serum AFP (ng/ml)**^**a**^		**RFS duration median**	20.4 (1–105.8 months)
≦200	115 (63.2%)	**Death**	
> 200	67 (36.8%)	No	130 (70.7%)
**Edmondson Grade**		Yes	54 (29.3%)
1 + 2	94 (51.1%)	**OS duration median**	44.4 (1–129.5 months)
3 + 4	90 (48.9%)		
**TNM stage**^**b**^			
I + II	106 (65.8%)		
III + IV	55 (34.2%)		

*HBV* hepatitis B virus, *HCV* Hepatitis C virus, *AFP* Alpha-fetoprotein, *TNM* Tumor-node-metastasis, *RFS* Relapse-free survival, *OS* Overall survival

^a^Data not available in 2 patients

^b^According to the American Joint Committee on Cancer (AJCC) TNM staging system 7th edition (2010) and Data not available in 23 patients

^c^Data not available in 23 patients. d:Relapse data not available in 4 patients

YULINK mRNA expression was measured by qRT-PCR, and its association with clinicopathological findings in HCC is presented in Table 2. Using the Youden index to determine the optimal cut-off values defining high and low expression groups, it was found that patients with high expression of YULINK were at higher risk than those with low expression for large tumor size, TNM III + IV stages, multiple tumors, vascular invasion, relapse, and death.

**Table 2:** Association of Yulink expression with clinical-pathological parameters in 184 patients with HCC

Variable	Yulink expression (*n* = 184)				
	**N**	**Low (*n* = 71)**	**High (*n* = 113)**	***P* value**^**a**^	**OR (95% CI)**
**Gender**					
Female	32	10	22	0.426	0.68 (0.3–1.53)
Male	152	61	91		
**Drinking history**					
Yes	56	18	38	0.254	1.49 (0.77–2.89)
No	128	53	75		
**HBV infection**					
negative	40	13	27	0.463	0.71 (0.34–1.49)
positive	144	58	86		
**HCV infection**					
negative	143	56	87	0.856	1.12 (0.54–2.29)
positive	41	15	26		
**Tumor size**					
≤ 5 cm	98	51	47	< **0.001**	3.58 (1.89–6.77)
> 5 cm	86	20	66		
**Serum AFP (ng/ml)**^**b**^					
≦200	115	49	66	0.211	1.52 (0.81–2.85)
> 200	67	22	45		
**Edmondson Grade**					
1 + 2	94	36	58	1	0.98 (0.54–1.77)
3 + 4	90	35	55		
**TNM stage**^**c**^					
I + II	106	52	54	**0.001**	3.45 (1.64–7.27)
III + IV	55	12	43		
**Vascular invasion**					
Absent	89	44	45	**0.004**	2.46 (1.34–4.53)
Present	95	27	68		
**Tumor Number**^**d**^					
Solitary	126	57	69	**0.007**	3.30 (1.34–8.12)
multiple	35	7	28		
**Cirrhosis**					
No	105	46	59	0.126	1.68 (0.91–3.10)
Yes	79	25	54		
**Metastasis**					
No	157	64	93	0.199	1.97 (0.79–4.92)
Yes	27	7	20		
**Relaspe**^**e**^					
No	73	47	26	** < 0.001**	6.60 (3.39–12.83)
Yes	107	23	84		
**Death**					
No	130	59	71	**0.004**	2.91 (1.40–6.03)
Yes	54	12	42		

Statistically significant values are displayed in boldface

*HBV* hepatitis B virus, *HCV* Hepatitis C virus, *TNM* Tumor-node-metastasis, *AFP* Alpha-fetoprotein, *OR* Odds Ratio

^a^Pearson Chi-square test

^b^Data not available in 2 patients

^c^Data not available in 23 patients

^d^Data not available in 23 patients

^e^Relapse data not available in 4 patients

**Fig. 1:**
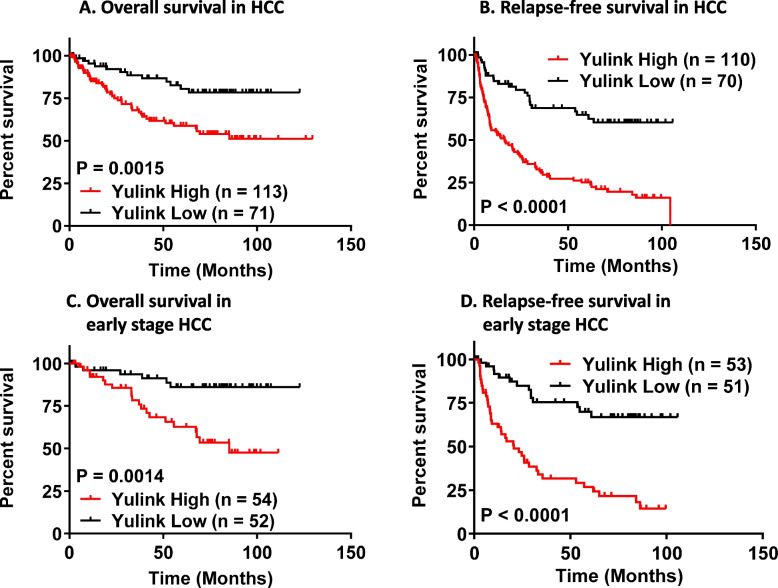
Kaplan–Meier plots of overall survival (OS) and relapse-free survival (RFS) for patients with HCC in relation to Yulink expression level. HCC patients with high level of Yulink had worse OS (**A**) and RFS (**B**). Early-stage HCC patients with high level of Yulink had worse OS (**C**) and RFS (**D**). Four out of 184 patients were excluded from relapse analyses due to the absence of relapse data

In addition, relapse-free survival (RFS) and overall survival (OS) of patients with HCC were analyzed using the Kaplan–Meier method. Patients with low YULINK expression in tumors had significantly higher OS (p = 0.0015) and RFS (P < 0.0001) than patients with high YULINK expression (Fig. 1A, 1 B). We then determined the potential prognostic value of YULINK expression in patients with early stage disease (stage 1–2). Early stage patients with low YULINK expression had significantly greater OS (*P* = 0.0014) and RFS (*P* < 0.0001) than patients with high level (Fig. 1C, 1D). Consistent with these findings, data mining using datasets from the Human Protein Atlas and The Cancer Genome Atlas (TCGA) showed that patients with HCC with high expression of *YULINK* had significantly shorter overall survival than those with low *YULINK* expression (P = 0.0079) (Supplementary Figure S1A). Further analysis also showed that Asian HCC patients with high *YULINK* expression exhibited significantly shorter overall survival than those with low *YULINK* expression (*P* = 0.0083) (Supplementary Figure S1B).

### YULINK expression as an independent prognostic factor for HCC

Univariate Cox proportional hazard regression analyses for RFS and OS are shown in Table 3, and RFS was correlated with tumor size ≥ 5 cm, multiple tumor numbers, Grade III + IV, vascular invasion, and metastasis. Furthermore, RFS was significantly associated with high *YULINK* expression in tumors. In addition, OS correlated with age ≥ 55 years, tumor size ≥ 5 cm, multiple tumor numbers, Grade III + IV, vascular invasion, metastasis, and high YULINK expression.

**Table 3:** Univariate and multivariable Cox proportional regression analysis of factors associated with HCC relapse and survival

Variables	RFS						OS					
	**Univariate analysis**			**Multivariable analysis**			**Univariate analysis**			**Multivariable analysis**		
	**HR**	**95% CI**	***P*-value**	**HR**	**95% CI**	***P*-value**	**HR**	**95% CI**	***P*-value**	**HR**	**95% CI**	***P*-value**
**Age: ≥ 55 vs. < 55**	1.35	0.91–1.99	0.13				**1.81**	**1.03–3.20**	**0.04**			NS
**Gender: male vs. female**	0.82	0.51–1.33	0.43				0.68	0.36–1.27	0.23			
**Hepatitis virus infection: yes vs. no**	0.77	0.31–1.88	0.56				1.08	0.26–4.44	0.91			
**Cirrhosis: present vs. absent**	1.09	0.74–1.59	0.67				1.04	0.61–1.78	0.89			
**Tumor size (cm): ≥ 5 vs. < 5**	**2.41**	**1.63–3.55**	** < 0.001**			NS	**3.16**	**1.81–5.53**	** < 0.001**	**2.10**	**1.11–3.99**	**0.02**
**AFP (ng/ml): ≥ 200 vs. < 200**	1.33	0.90–1.97	0.15				1.63	0.95–2.80	0.08			
**Tumor number: multiple vs. solitary**	**2.01**	**1.29–3.13**	**0.002**			NS	**2.97**	**1.72–5.14**	** < 0.001**			NS
**Grade: III + IV vs I + II**	**2.13**	**1.42–3.20**	** < 0.001**			NS	**2.76**	**1.61–4.74**	** < 0.001**			NS
**Vascular invasion: present vs. absent**	**2.89**	**1.93–4.31**	** < 0.001**	**2.03**	**1.24–3.31**	** < 0.001**	**4.16**	**2.22–7.79**	** < 0.001**	**2.28**	**1.09–4.76**	**0.03**
**Metastasis: yes vs. no**	**2.81**	**1.79–4.41**	** < 0.001**	**1.87**	**1.11–3.15**	**0.018**	**2.27**	**1.21–4.26**	**0.01**			NS
**Tumor Yulink: high vs. low**	**3.43**	**2.16–5.46**	** < 0.001**	**2.63**	**1.58–4.38**	** < 0.001**	**2.72**	**1.43–5.17**	**0.002**			NS

Multivariable modeling using stepwise variable selection. Statistically significant values are displayed in boldface

*RFS* Relapse-free survival, *OS* Overall survival, *HR* Hazard ratio, *CI* Confidence interval, *NS* Not significant

Next, to identify independent variables associated with poor RFS or OS, we selected these covariates for multivariate Cox regression analysis in a stepwise manner (Table 3). Metastasis and vascular invasion were found to be independent risk factors for RFS. Notably, the expression of *YULINK* in tumors was an important and independent risk factor for RFS (HR, 2.63; 95% CI: 1.58–4.38, *P* < 0.001). But for OS, only tumor size and vascular invasion were independent risk factors for OS.

### Effects of YULINK expression in cell migration under 1 or 25 mM glucose condition

**Fig. 2:**
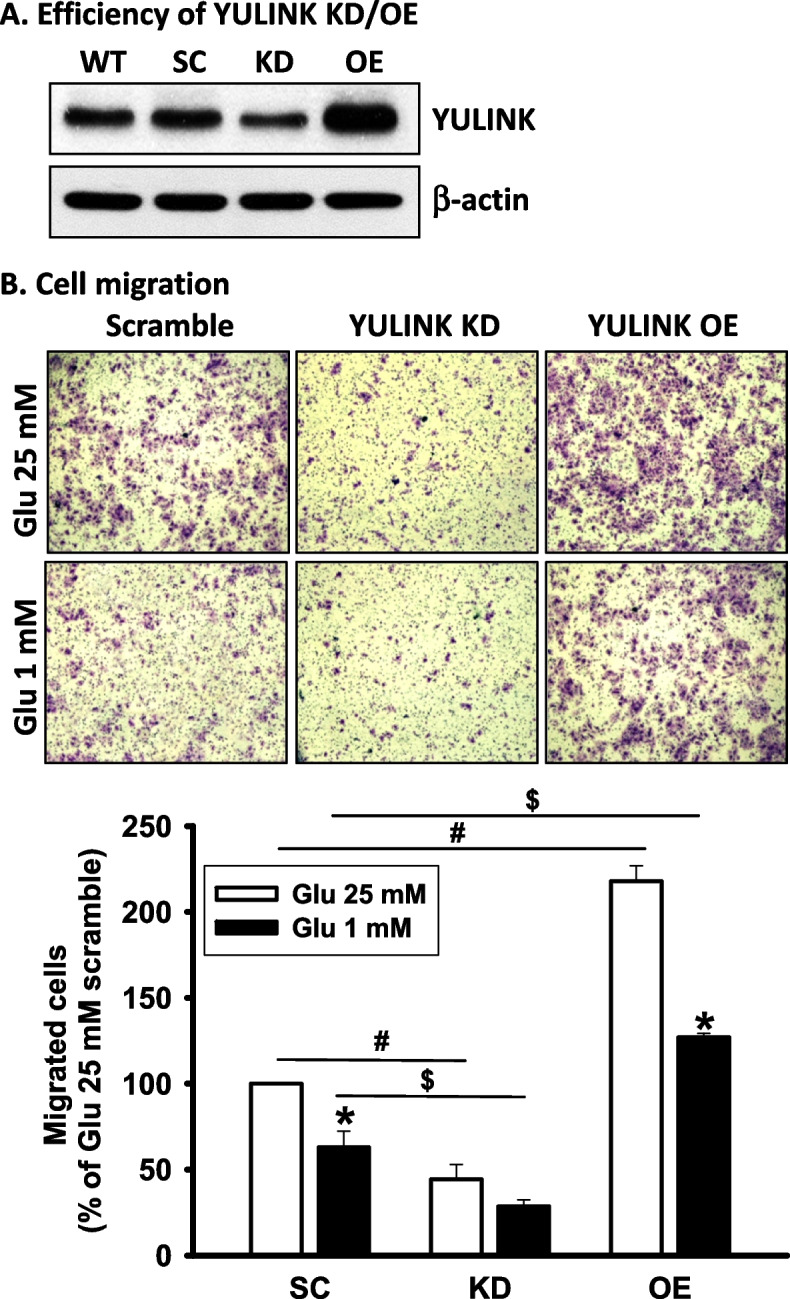
The positive correlation between YULINK expression and cell migration. **A** YULINK expression in wild-type (WT), scramble control (SC), YULINK knockdown (KD), and YULINK OE Huh7 cells was examined by Western blot analysis. -actin levels were used as loading controls. **B** Representative micrographs depict cell migration in Huh7 cell with YULINK knockdown or overexpression under 25 mM or 1 mM glucose conditions by transwell analysis. Magnification: 400 ×. Presented values of bar graphs represent the mean of three independent experiments ± S.D. *P < 0.05 indicates significant differences between glucose 25 mM and 1 mM within the YULINK SC, KD, and OE group. # and $P < 0.05 indicate significant differences between the indicated comparison groups

Since YULINK expression is associated with advanced HCC, including vascular invasion and metastasis (Table 3), it was suggested that YULINK expression might affect the migration of Huh7 cells. Therefore, Huh7 cells with either YULINK knockdown or overexpression were prepared and confirmed by Western blot analysis (Fig. 2A). Migration analysis further demonstrated that Huh7 cell migration is positively correlated with both glucose concentration and YULINK expression (Fig. 2B). Upon YULINK knockdown, Huh7 cell migration was suppressed in either 1 or 25 mM glucose culture media. On the other hand, cell migration was increased in either 1 or 25 mM glucose conditions when YULINK was overexpressed in cells.

### YULINK knockdown reduced cell viability and enhanced cell death under glucose restriction conditions

**Fig. 3:**
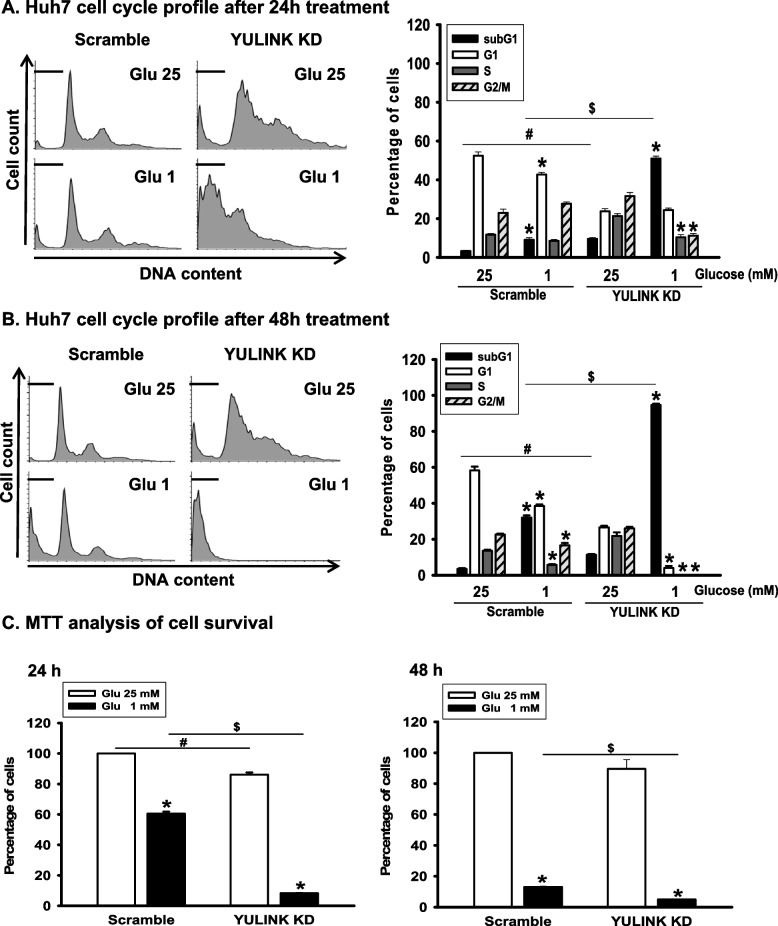
Loss of YULINK enhanced glucose restriction-triggered cell death. Huh7 cells with or without YULINK knockdown were maintained under 25 mM glucose or glucose restriction conditions for (**A**) 24 h or (**B**) 48 h before harvesting. The cell cycle distribution was analyzed by flow cytometry. In each panel, cell cycle profiles are presented together with bar graphs, indicating the cell distribution in each phase of the cell cycle. Values in the bar graphs represent the mean of three independent experiments ± S.D. *P < 0.05 indicates significant differences between glucose 25 mM and 1 mM within the Scramble and YULINK KD group. # and $P < 0.05 indicate significant differences between the indicated comparison groups. (C) MTT analysis of Huh7 cells with or without YULINK knockdown cultured with 25 mM glucose or glucose restriction conditions for the indicated times. Bar graphs represent the mean of three independent experiments ± S.D. *P < 0.05 indicates significant differences between glucose 25 mM and 1 mM within the Scramble and YULINK KD group. # and $P < 0.05 indicate significant differences between the indicated comparison groups

Next, we examined whether YULINK knockdown led to a death response in glucose-restricted cells. Huh7 cells were cultured in a medium containing 25 or 1 mM glucose with or without YULINK knockdown for up to 48 h and analyzed for DNA content using flow cytometry (Fig. 3). Huh7 scramble control cells under glucose restriction for 24 h exhibited a DNA profile with a decreased G1 phase and an increased subG1 population compared to cells treated with 25 mM glucose (Fig. 3A). In comparison, YULINK knockdown cells exhibited an accumulation of cells arrested in the S and G2/M phases and a significant increase in the subG1 population (to about 10% of cells) compared to the scramble control under 25 mM glucose. In contrast, under glucose restriction for 24 h, YULINK knockdown cells showed a robust apoptotic response, with over 50% of cells in the subG1 population (Fig. 3A), whereas elevated level of subG1 DNA content to almost 100% were observed after 48 h of glucose restriction (Fig. 3B). Consistently, HA22T cells, another highly invasive HCC cell line commonly used in chemo-resistance studies also exhibited a significantly higher subG1 population following YULINK knockdown than scramble controls under 12-h glucose restriction conditions (Supplementary Figure S2). The results of the MTT assay confirmed that cells with YULINK knockdown had a survival of approximately 10% as compared to 60% survival of its scramble control under glucose restriction for 24 h. Cell survival decreased even further after 48 h of glucose restriction. Notably, even under normal glucose conditions, cell survival was still reduced in YULINK knockdown cells (Fig. 3C).

### Increased DNA damage and reactive oxygen species (ROS) responses in Huh7 cells with YULINK knockdown under glucose-restricted condition

**Fig. 4:**
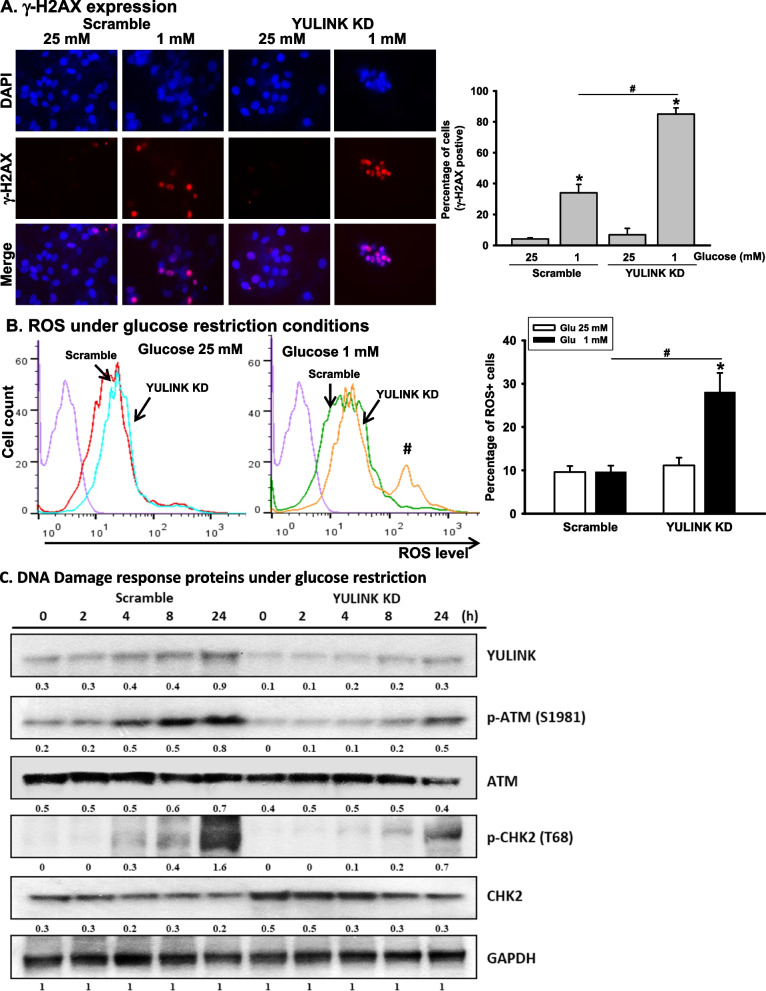
Loss of YULINK enhanced γ-H2AX expression, ROS production, and failure to trigger ATM-CHK2 activation in Huh7 cells under glucose restriction.** A** Huh7 cells with or without YULINK knockdown were maintained under 25 mM glucose or glucose restriction for 24 h before fixing and staining for γ-H2AX pSer139. The percentage of cells (blue) and γ-H2AX (red) signals were counted and are presented in the bar graphs. Values represent the mean of three independent experiments ± S.D. *P < 0.05 indicates significant differences between glucose 25 mM and 1 mM within the Scramble and YULINK KD group. #P < 0.05 indicates significant difference between the indicated comparison groups. **B** Huh7 cells with or without YULINK knockdown were maintained under 25 mM glucose or glucose restriction conditions for 24 h before being harvested and subjected to ROS analysis using flow cytometry. The arrowhead indicates an increase in ROS. The cell populations in areas having different ROS levels were determined using FlowJo software. The results are presented together with bar graphs, indicating the percentage of cells that were ROS +. Values represent the mean of three independent experiments ± S.D. *P < 0.05 indicates significant differences between glucose 25 mM and 1 mM within the Scramble and YULINK KD group. #P < 0.05 indicates significant difference between the indicated comparison groups. (C) Huh7 cells with or without YULINK knockdown were maintained under glucose restriction (1 mM) for 0–24 h and harvested for Western blot analysis for indicated proteins at the times shown. GAPDH served as internal control. The numbers below the blots represent normalization against GAPDH

Histone H2AX is generally considered a marker of DNA damage because it is phosphorylated in response to DNA damage and recruited to damage sites where breaks of particular DNA strands occur (Contreras et al. 2024). Since an increase in cell death was noted in YULINK knockdown Huh7 cells under glucose restriction, DNA damage was examined by immunofluorescence assay. The red fluorescent signals in Fig. 4A represent the expression of pSer139 H2AX (γ-H2AX) in Huh7 cells. Under glucose restriction conditions, more than 80% of Huh7 cells with YULINK knockdown were positive for γ-H2AX (γ-H2AX +) as compared to the scramble controls (about 40% γ-H2AX +). The finding that increased γ-H2AX + expression in cells indicated extensive DNA damage in YULINK knockdown Huh7 cells under glucose restriction. In contrast, no notable enhancement in γ-H2AX expression was observed in YULINK overexpressing cells even under glucose restriction. Instead, there was a decrease in γ-H2AX levels, suggesting that YULINK may play a protective role in response to stress induced by glucose deprivation (Supplementary Figure S3).

It has been reported that metabolic stress such as glucose starvation triggers cell death by promoting ROS production and cytotoxicity (Kang et al. 2023). Given the increased DNA damage and cell death response following glucose restriction in YULINK knockdown cells, we examined whether these responses were related to increased oxidative stress. As shown in Fig. 4B, an increase in ROS production was observed in YULINK knockdown cells compared to scramble controls in 1 mM glucose. This indicated that DNA damage and the cell death response following glucose restriction in YULINK knockdown cells were associated with oxidative stress.

In mammalian cells, there are two conserved signalling cascades, ATM-CHK2 and ATR-CHK1, which delay or arrest cell cycle progression, thus allowing time for damage repair or overcoming stress under unfavorable conditions (Smith et al. 2020). However, if these checkpoints are deregulated, genomic instability would occur and contribute to cancer progression (Matthews et al. 2022). In addition to the activation of genotoxic stress and DNA damage repair, checkpoints are activated in response to nutrient starvation, which causes metabolic stress. Therefore, we examined whether the ATM-CHK2 pathway was activated following DNA damage caused by glucose restriction in Huh7 cells, with or without YULINK knockdown. The ATM-CHK2 pathway is activated by DNA double-strand breaks with the phosphorylation of ATM at pSer 1981 and CHK2 at pThr68, which leads to cell cycle arrest and stimulation of DNA repair (Phan and Rezaeian 2021). First, scramble control and YULINK knockdown cells were treated with glucose restriction and harvested at different time points for Western blot analysis. As shown in Fig. 4C, activation of pSer1981 ATM phosphorylation began to increase at 4 h in scramble control Huh7 cells and persisted for up to 24 h under glucose restriction conditions. Enhanced expression of the pThr68 CHK2 protein was also observed, reaching peak levels at 24 h. In comparison, although total CHK2 levels were slightly increased in YULINK knockdown cells compared to their scramble control counterparts, the glucose restriction-induced enhancement of phosphorylated ATM and phosphorylated CHK2 was reduced in YULINK knockdown cells (Fig. 4C). These results suggest that the glucose restriction-induced ATM-CHK2 pathway was not fully activated in YULINK knockdown cells, which might correlate with increased cell death under glucose restriction.

### YULINK overexpression decreased glucose restriction-caused cell death in Huh7 cells

**Fig. 5:**
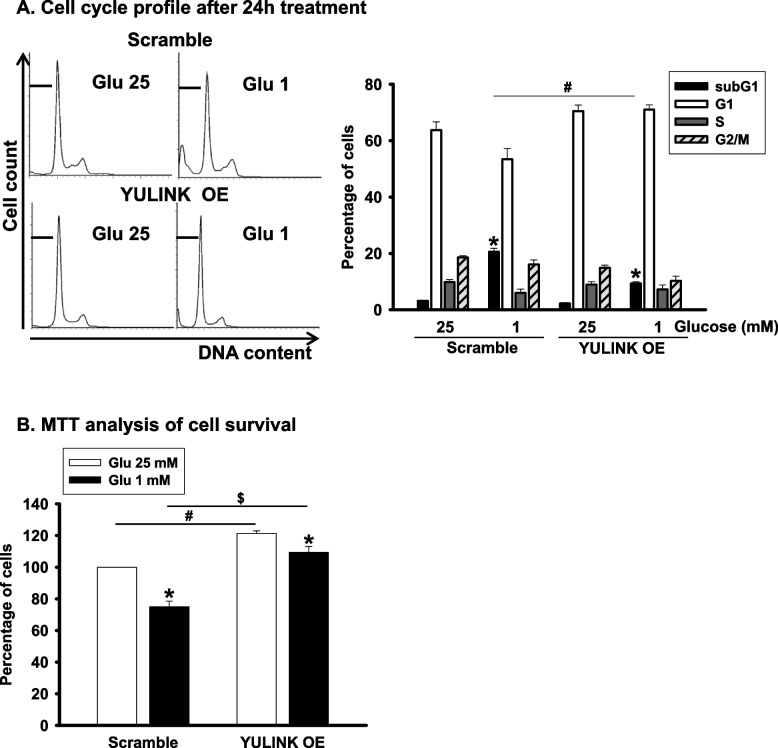
YULINK overexpression enhanced cell migration and rescued cell death in Huh7 cells under glucose restriction condition. **A** Huh7 cells with or without YULINK overexpression were maintained under 25 mM glucose or glucose restriction conditions for 24 h before harvesting. The cell cycle distribution was analyzed by flow cytometry. Cell cycle profiles are presented together with bar graphs, indicating the cell distribution in each phase of the cell cycle. Values in the bar graphs represent the mean of three independent experiments ± S.D. *P < 0.05 indicates significant differences between glucose 25 mM and 1 mM within the Scramble and YULINK OE group. #P < 0.05 indicates significant difference between the indicated comparison groups. **B** MTT analysis of Huh7 cells with or without YULINK overexpression cultured with 25 mM glucose or glucose restriction conditions for the indicated times. Bar graphs represent the mean of three independent experiments ± S.D. *P < 0.05 indicates significant differences between glucose 25 mM and 1 mM within the Scramble and YULINK OE group. # and $P < 0.05 indicates significant difference between the indicated comparison groups

To further confirm the positive correlation between cell survival and YULINK expression, especially under glucose restriction conditions, we investigated whether glucose restriction-induced cell death could be decreased in Huh7 cells overexpressing YULINK. Huh7 cells with or without YULINK overexpression were cultured with 25 or 1 mM glucose for 24 h and subjected to flow cytometry analysis to examine the DNA content. As shown in Fig. 5A, the subG1 population was significantly decreased in Huh7 cells overexpressing YULINK under glucose restriction for 24 h as compared to the scramble controls. Furthermore, MTT analysis showed increased Huh7 survival and cell proliferation in YULINK overexpressing Huh7 cells under glucose restriction for 24 h (Fig. 5B). Taken together, these results demonstrated that YULINK plays a protective role in Huh7 cells under glucose restriction, as overexpression of YULINK was associated with decreased cell death and increased cell survival.

### *In vivo* effects of YULINK knockdown in Huh7 xenografts

**Fig. 6:**
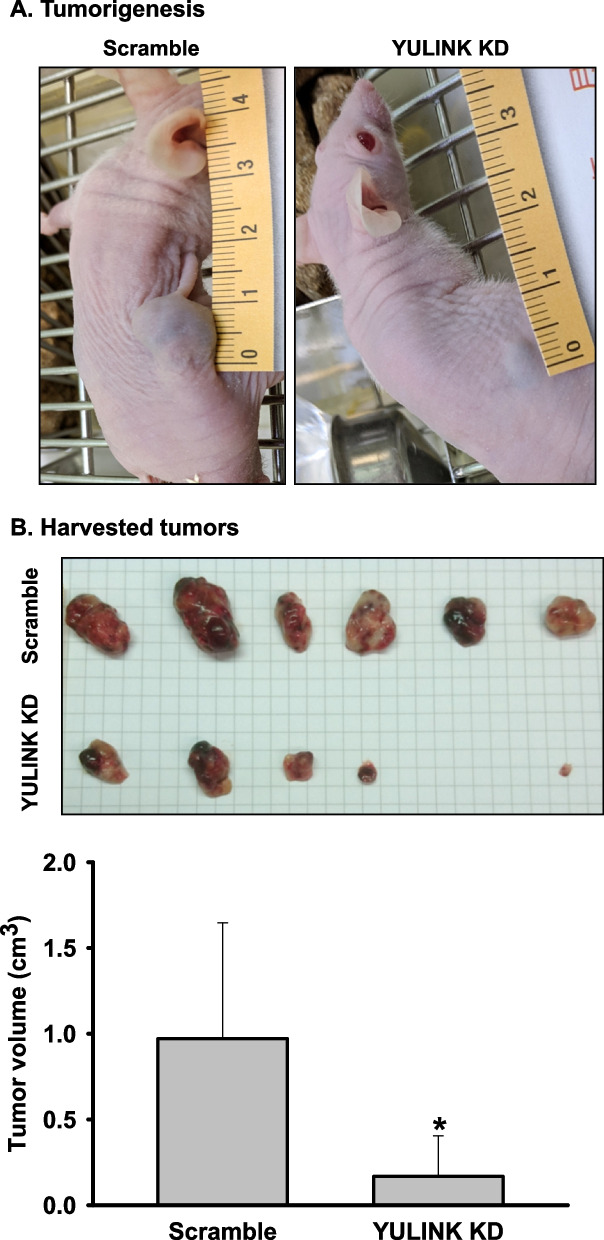
YULINK deficiency in Huh7 cells delays tumor formation in a xenograft mouse model. A Huh7 cells with or without YULINK knockdown were subcutaneously injected into the dorsal region of 6-week-old nude mice and monitored for tumor formation for a period of 8 weeks. SC group: *n* = 7; KD group: *n* = 8. **B** Representative image and accompanying bar graph show that six out of seven mice injected with scramble control Huh7 cells developed visible tumors (one mouse died in the second week post-injection). In contrast, only five mice in the YULINK knockdown group (*n* = 5/8) exhibited tumor formation, with significantly smaller tumor sizes. *P < 0.05 indicates significance between compared groups

Additionally, we investigated the role of YULINK in HCC tumorigenesis in vivo. Nude mice were divided into two groups and subcutaneously injected with Huh7 cells with or without the YULINK knockdown for tumorigenesis (Fig. 6A). After eight weeks, the mice were sacrificed, and tumor formation was determined in six out of seven nude mice in the scramble control group. However, in YULINK knockdown Huh7 cells, only five mice with significantly smaller tumor sizes were recognized (*n* = 5/8) (Fig. 6B). These results indicated that xenografts with YULINK knockdown inhibited tumor progression. Taken together, our results revealed that YULNK plays an important role in the tumorigenesis of Huh7 xenografts in vivo.

### YULINK knockdown decreased glucose uptake and glycolysis in Huh7 cells

**Fig. 7:**
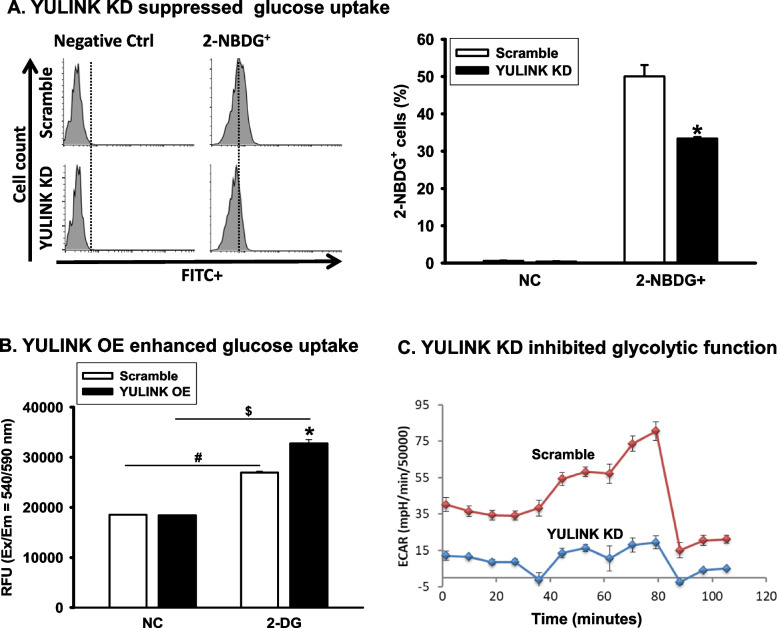
YULINK expression positively associated with glucose uptake and glycolysis. A Huh7 cells with or without YULINK knockdown were treated with 2-NBDG for 15 min and subjected to flow cytometric analysis. Bar graphs illustrate the percentage of Huh7 cells taking up 2-NBDG. Values represent the mean ± S.D. of three independent experiments. *P < 0.05 indicates significant differences between the Scramble and YULINK KD group. **B** Huh7 cells with or without YULINK overexpression were incubated with 2-DG for 30 min and analyzed using a fluorescence microplate reader at excitation/emission wavelengths of 540/590 nm. RFU: relative fluorescence unit. *P < 0.05 indicates significant differences between the Scramble and YULINK OE group. # and $P < 0.05 indicates significant difference between the indicated comparison groups. (C) Extracellular acidification rate of Huh7 cells with or without YULINK knockdown

To determine whether the effect of YULINK on cell fate in HCC is related to glucose metabolism, we first examined how YULINK influences glucose uptake. Huh7 cells with or without YULINK knockdown were subjected to glucose uptake assay using non-catabolized 2-NBDG as a fluorescent deoxyglucose analog. While 2-NBDG was detected in more than 50% of cells in the scramble control, only about 30% of cells with YULINK knockdown were detected with 2-NBDG (Fig. 7A). In contrast, when YULINK was overexpressed in Huh7 cells, the cells exhibited a significant increase in glucose uptake in 2-DG glucose uptake analysis (Fig. 7B). These results indicated a positive correlation between YULINK levels and glucose uptake in Huh7 cells.

Since glucose uptake is one of the key components of the glycolytic pathway, we examined the role of YULINK in cellular anaerobic glycolysis. Glycolysis produces pyruvate, which is converted to lactate under hypoxic or partially anaerobic conditions. Thus, the extracellular acidification rate could reflect the status of functional glycolysis (Zhang and Zhang 2019). As shown in Fig. 7C, a substantial decrease in the extracellular acidification rate was observed in Huh7 cells with YULINK knockdown relative to that in scramble controls. Altogether, these results indicate that YULINK knockdown not only decreased glucose uptake but also disrupted functional glycolysis in Huh7 cells. Similar results were observed in HA22T cells (Supplementary Figure S4A, S4B).

### YULINK knockdown suppressed the expression of GLUT1 and its translocation

**Fig. 8:**
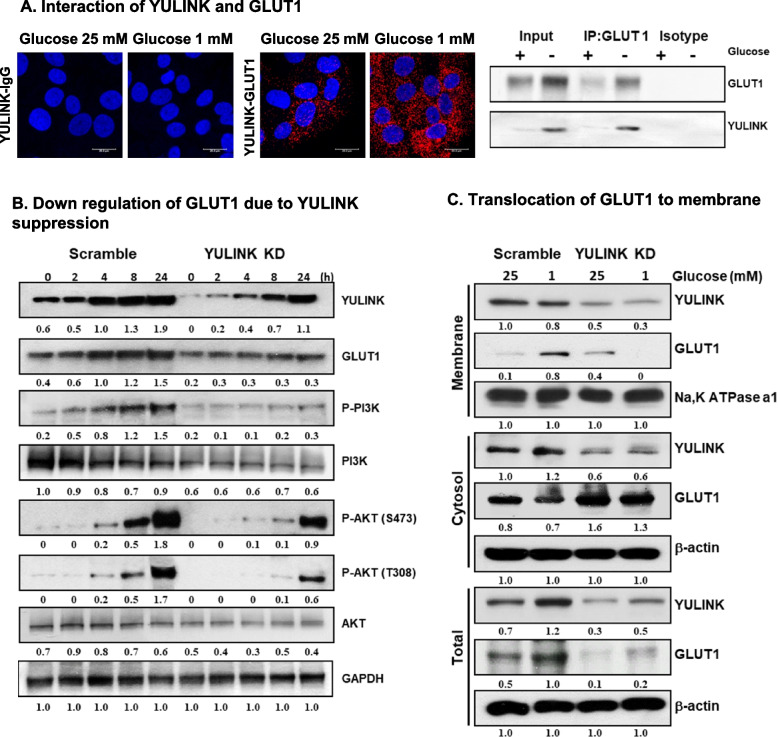
YULINK suppression decreased glucose restriction-induced GLUT1 expression and translocation. A Huh7 cells with or without YULINK knockdown were cultured with 25 mM or 1 mM glucose conditions before being fixed and examined by proximity ligation assay analysis. The red spots in images represent positive YULINK-GLUT1 interactions. The blue areas indicate DAPI-stained nucleus (left panel). In addition, cell lysates derived from Huh7 cells cultured with normal glucose (25 mM) or glucose restriction (1 mM) condition were harvested and incubated with anti-GLUT1-coated magnetic beads. YULINK and GLUT1 expression in the immunoprecipitates were further determined by using Western blot analysis (right panel). **B** Huh7 cells with or without YULINK knockdown were maintained under glucose restriction conditions (1 mM) for 0–24 h before Western blot analyses for the indicated proteins. GAPDH served as internal control. The numbers below the blots represent normalization against GAPDH. **C** Membrane, cytosol and total protein lysates were prepared from Huh7 cells with or without YULINK knockdown cultured with 25 mM or 1 mM glucose conditions for 24 h and subjected to Western blot analyses for the indicated proteins. Na/K ATPase α1 and β-actin served as loading controls. The numbers below the blots represent normalization against internal control

GLUT1 is a key rate-limiting factor for glucose uptake and glycolysis in HCC cells (Mossenta et al. 2020). To analyze whether the observed decrease in glucose uptake after YULINK knockdown was associated with GLUT1, we examined the interaction between YULINK and GLUT1 using a proximity-ligation assay. The signal in the proximity ligation assay (red) is indicative of an interaction event. As shown in Fig. 8A, proximity ligation assay signals were found in Huh7 cells and further increased under glucose restriction conditions. This YULINK-GLUT1 interaction was also confirmed by immunoprecipitation analysis, where YULINK was detected by Western blotting in protein lysates immunoprecipitated with an anti-GLUT1 antibody (Fig. 8A, right).

To determine whether GLUT1 expression was affected by YULINK expression, Western blotting was performed to assess the expression of YULINK and GLUT1 in Huh7 cells at different time points under glucose restriction for up to 24 h (Fig. 8B). As shown in Fig. 8B, the expression of YULINK and GLUT1 in scramble control cells increased under glucose restriction conditions. However, when YULINK was knocked down, the expression of GLUT1 decreased under either 25 mM glucose (0 h) or glucose restriction conditions (2–24 h). It is known that GLUT1 expression and translocation are regulated by AKT and inhibited by PI3K inhibitors (Mossenta et al. 2020; Hsieh et al. 2019). Glucose restriction is also known to suppress energy-dependent pathways, including PI3K-Akt and mTOR signalling in cancer cells (Kim et al. 2019). We next examined whether GLUT1 expression affected by YULINK knockdown was associated with PI3K-AKT signaling under glucose restriction conditions. As shown in Fig. 8B, the phosphorylation of PI3K and AKT were both decreased in YULINK knockdown Huh7 cells compared to the scramble control.

Furthermore, to examine whether the translocation of GLUT1 was affected by YULINK expression, the expression of GLUT1 in the membrane and cytosolic fractions of Huh7 cells under 25 mM glucose or glucose restriction for 24 h was analyzed separately. In scramble control cells, GLUT1 expression in the membrane fraction was higher and its expression in the cytosolic fraction was lower under glucose restriction conditions than in 25 mM glucose (Fig. 8C). In contrast, in YULINK knockdown Huh7 cells, GLUT1 expression in the membrane fraction was lower under glucose restriction than in 25 mM glucose (Fig. 8C). In addition, in the cytosolic fraction, GLUT1 expression was higher in YULINK knockdown cells than in scramble controls under both 25 mM glucose and glucose restriction conditions (Fig. 8C). Furthermore, decreased GLUT1 expression was found in total cell lysates of YULINK knockdown Huh7 cells compared to scramble controls under both 25 mM glucose and glucose restriction conditions. These findings suggest that GLUT1 expression and translocation are affected by YULINK in Huh7 cells, especially under glucose-restricted conditions.

## Discussion

In this study, we demonstrated that YULINK expression was an independent prognostic factor in patients with HCC. In addition, our in vitro and in vivo experiments showed that YULINK deficiency inhibited tumor proliferation. Enhanced cell death could be associated with increased DNA damage response and ROS in YULINK knockdown Huh7 cells under glucose restriction conditions. Furthermore, YULINK expression was correlated with glucose uptake and glycolysis through GLUT1 regulation and protein interaction. These findings suggest that YULINK is involved in glucose metabolism in HCC cells.

Cox regression analysis demonstrated that patients with tumors with high *YULINK* expression had significantly worse OS and RFS than those with *YULINK*-low tumors. Multivariate analysis revealed *YULINK* expression was an independent factor for HCC relapse, suggesting that *YULINK* higher or lower levels is prognostic factors for HCC. To the best of our knowledge, this is the first report to support *YULINK* expression as an independent prognostic factor in cancers.

Deregulated cell growth and metabolism significantly contributes to cancer development and progression. It has been reported that the common glucose transporter in humans, GLUT1, is overexpressed in malignancies including hepatic, pancreatic, breast, brain, renal, lung, colorectal, ovarian, and cervical cancers (Yu et al. 2017). HCC cells reprogram glucose metabolism to fulfil their anabolic demands via GLUT1 overexpression, which differs from normal hepatocytes (Lei et al. 2020). GLUT1 elevation also indicates poor prognosis for HCC in terms of increased invasiveness and metastasis (Mossenta et al. 2020). In this study, the expression and function of GLUT1 were suppressed when YULINK was suppressed in Huh7 cells under either 25 or 1 mM glucose conditions. Furthermore, our results revealed that low YULINK expression in patients with HCC is associated with significantly longer survival. Our novel findings imply that YULINK may be involved in the regulation of GLUT1 expression in HCC cells.

In addition, Bidkhori *et. al.* recently identified three HCC subtypes with different metabolic and signaling pathways using a genome-scale metabolic network (Bidkhori et al. 2018). These subtypes showed large differences in clinical survival associated with altered kynurenine metabolism (iHCC1), WNT/β-catenin–associated lipid metabolism (iHCC2), and PI3K/AKT/mTOR signaling (iHCC3). Kaplan–Meier survival analysis showed significant differences in patient survival among the three HCC subtypes (iHCC1 > iHCC2 > iHCC3). iHCC3 cells showed higher glycolytic but lower citric acid cycle fluxes, which was consistent with a strong Warburg effect. As shown in Supplementary Figure S5, YULINK FPKM was significantly higher in iHCC3 than in iHCC1 (*P* < 0.05) and iHCC2 (*P* < 0.01). These findings are compatible with our results that YULINK might be associated with PI3K/AKT signaling in terms of glucose metabolism in HCC.

The concentrations of certain nutrients (e.g., glucose) in solid tumors are lower than those in normal tissues owing to their high cellularity, increased glycolytic rate, and insufficient vasculature. Thus, cancer cells adapt their metabolic functions to unfavorable microenvironments, such as glucose restriction (Wang et al. 2020). In our study, increased death of YULINK knockdown Huh7 cells under conditions of glucose restriction was demonstrated. Delayed tumorigenesis in immunocompromised nude mice due to xenografts with YULINK knockdown was also confirmed in vivo. Furthermore, YULINK knockdown either decreased GLUT1 expression or inhibited GLUT1 translocation under glucose-restricted conditions, resulting in suppression of glucose uptake and glycolysis. Taken together, our in vitro and in vivo data demonstrate the crucial role of YULINK in Huh7 cells in maintaining glucose metabolism for survival, particularly under glucose restriction conditions.

The starvation of cancer cells may be a possible strategy for decreasing glucose uptake. In addition, GLUT1 is a target for aerobic glycolysis inhibition in various cancer therapies (Li et al. 2023; Wu et al. 2020). In our study, under glucose restriction conditions (1 mM), increased GLUT1 expression was found in total cell lysates and the membrane fraction of Huh7 cells, implying that glucose restriction prompted GLUT1 translocation from cytosol to membranes in Huh7 cells, as indicated by the lower expression of GLUT1 in the cytosolic fraction compared to its expression in 25 mM glucose. Our findings are consistent with a previous report that GLUT1 is synthesized and translocated from the cytoplasm to the membranes in culture medium with low glucose concentration in 3T3L1 cells (Crone et al. 2000). Under glucose restriction conditions, diminished membrane GLUT1 and increased cytosolic GLUT1 were found in YULINK knockdown Huh7 cells compared with scramble controls. In addition, the expression of GLUT1 in total lysates of YULINK knockdown Huh7 cells decreased under glucose restriction conditions. Taken together, the expression and translocation of GLUT1 were inhibited in YULINK knockdown Huh7 cells under glucose restriction, suggesting an important role of YULINK in GLUT1 regulation.

Metabolic stress resulting from glucose restriction can lead to ROS-induced cytotoxicity caused by ROS (Ghanbari Movahed et al. 2019). ROS are believed to cause base substitution mutations, which in turn lead to double-stranded DNA breaks. Cancer cells with a high metabolic rate have higher levels of ROS than normal cells, which makes them vulnerable to ROS-induced cell death (Ghanbari Movahed et al. 2019; Perillo et al. 2020). In this study, an increase in ROS was noted in YULINK knockdown Huh7 cells under glucose restriction compared with scramble controls. The increase in ROS resulting from metabolic stress in YULINK knockdown Huh7 cells under glucose restriction may account for the observed increase in DNA damage. In addition, inactivation of ATM-CHK2 signaling, which is associated with DNA damage, further enhanced cell death in YULINK knockdown Huh7 cells. It has been reported that ATM-CHK2 signaling plays an important role in DNA damage responses, enabling cells to survive stress conditions, particularly DNA double-stranded breaks (Phan and Rezaeian 2021; Menolfi and Zha 2020). Following DNA damage, ATM-CHK2 is activated to phosphorylate substrates that regulate DNA repair, the cell cycle, and apoptosis. However, when YULINK was downregulated under glucose restriction conditions in Huh7 cells, cell death increased, which was accompanied by unrepaired DNA damage (e.g., as indicated by γ-H2AX activation) and inactivation of ATM-CHK2 signaling. These results implied that YULINK may play a role in ATM-CHK2 activation. However, the precise molecular mechanism underlying this regulation remains unclear. This represents a limitation of our current study and warrants further investigation in future research to delineate how YULINK modulates the DNA damage response pathway.

## Conclusions

**Fig. 9:**
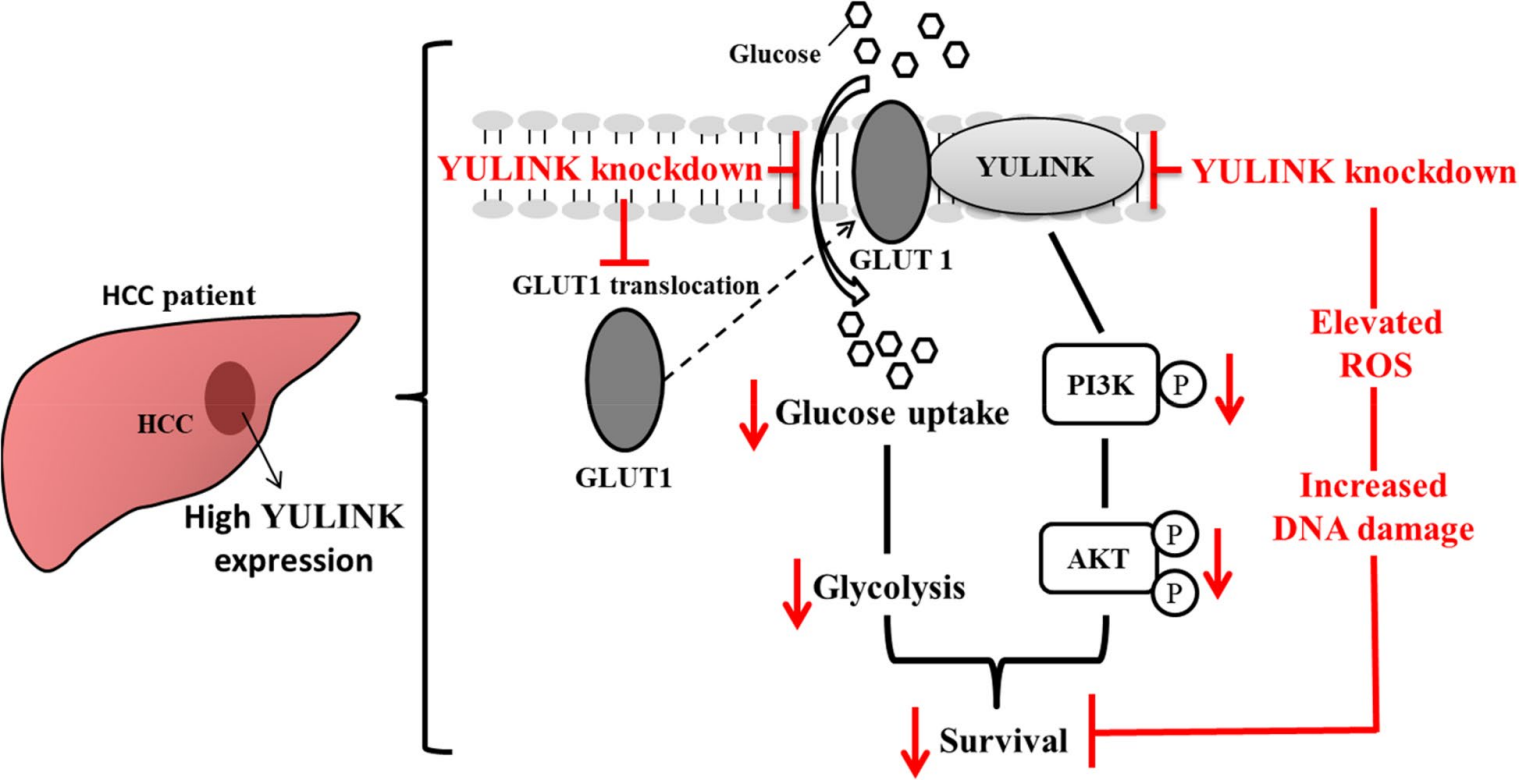
Putative mechanism elucidating how YULINK facilitates cancer cell survival in hepatocellular carcinoma (HCC). A high level of YULINK in HCC patients is associated with poor prognosis in clinicopathologic analysis. Within the naturally low-glucose tumor microenvironment, YULINK plays a pivotal role in regulating glucose uptake and glycolysis by interacting with GLUT1 and participating in the PI3K-AKT pathway. YULINK knockdown not only disrupts GLUT1 translocation, glucose uptake, glycolysis, and PI3K-AKT signaling, but also impairs the DNA damage response, leaving cells unable to protect themselves from elevated reactive oxygen species (ROS), resulting in DNA damage and eventual cell death. The red color denotes the outcome associated with YULINK deficiency

In summary, our study highlights a positive correlation between YULINK and poor prognosis in patients with clinical HCC, emphasizing its crucial role in HCC survival. While glucose restriction induced the upregulation of both YULINK and GLUT1 in Huh7 cells, YULINK deficiency disrupted GLUT1-dependent glucose uptake and functional glycolysis, which are particularly essential under glucose deprivation conditions and could be associated with the PI3K-AKT pathway. Although our findings support a potential link between YULINK and GLUT1-mediated glycolytic regulation, the precise mechanistic contributions of GLUT1 to YULINK-dependent phenotypes warrant further investigation. Follow-up studies, including targeted modulation of GLUT1 expression in the context of YULINK knockdown or overexpression, will help clarify the extent of this interaction and uncover additional downstream effectors involved. Functionally, HCC cells lacking YULINK exhibited increased cell death in response to glucose restriction in vitro and delayed tumor progression in vivo. Furthermore, elevated ROS levels and inactivation of protective ATM-CHK2 signaling were found in YULINK knockdown Huh7 cells under glucose restriction. These factors could also contribute to the increased DNA damage and consequent cell death observed in the YULINK-deficient cells. Conversely, enhanced cell death was reduced in Huh7 cells overexpressing YULINK even under glucose-restricted conditions. These findings are compatible with our clinical pathologic analysis of HCC, which revealed that low expression of YULINK in HCC was associated with better survival (Fig. 9).

## Supplementary Information

Supplementary Material 1.

## Data Availability

No datasets were generated or analysed during the current study.

## Abbreviations

HCC: Hepatocellular carcinoma
GLUT1: Glucose transporter isoform 1
ATM: Ataxia telangiectasia mutated
CHK2: Checkpoint kinase 2
ROS: Reactive oxygen species
GLUT2: Glucose transporter isoform 2
mio: Missing oocyte
mTOR: Mammalian target of rapamycin
ATR, ATM and RAD3-related: CHK1, checkpoint kinase 1
FPKM: Fragments per kilobase of exon per million reads
OE: Overexpression
ROC: Receiver operating characteristic
DMEM: Dulbecco's modified Eagle's medium
FBS: Fetal bovine serum
shRNA: Short hairpin RNA
2-NBDG: 2-Deoxy-2-[(7-nitro-2,1,3-benzoxadiazol-4-yl) amino]-D-glucose
ECAR: Extracellular acidification rate
MTT: 3-(4,5-Dimethylthiazol-2-yl)-2,5-diphenyltetrazolium bromide
DMSO: Dimethyl sulfoxide
PMSF: Phenylmethylsulphonyl fluoride
SDS-PAGE: Sodium dodecyl sulfate–polyacrylamide gel electrophoresis
PI3K: Phosphatidylinositide 3-kinases
AKT: Protein kinase B
S.D.: Standard deviation
KD: Knockdown
Ctrl: Control
Glu: Glucose
RFS: relapse-free survival
OS: overall survival
