# Supplementary material for: YULINK deficiency promotes cell death under glucose restriction in HCC cells in association with GLUT1-mediated glycolysis
Source: Mol Med. 2025 Sep 26;31:291. doi: 10.1186/s10020-025-01347-7 (PMC12465497; doi:10.1186/s10020-025-01347-7)
Supplement: Supplementary file 1 — Supplementary Material 1. [file 10020_2025_1347_MOESM1_ESM.docx]

**Supplementary Figure S1**

**
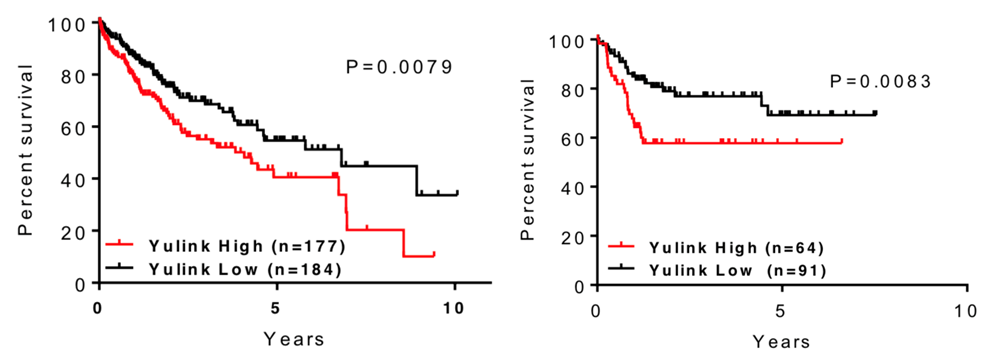
**

**YULINK expression in patients with liver cancer.** A) The expression of YULINK was statistically analyzed in patients with hepatocellular carcinoma (HCC). The association between YULINK expression level and survival was also analyzed in B) Asian patients with HCC and high YULINK expression (mean survival: 4.108, 95% CI: 3.333–4.882 years) and low YULINK expression (mean survival: 5.776, 95% CI: 5.101–6.451 months). The raw data were obtained from the Human Protein Atlas (website: www.proteinatlas.org) and The Cancer Genome Atlas (TCGA) database. (website: https://cancergenome.nih.gov)

**Supplementary Figure S2**

**
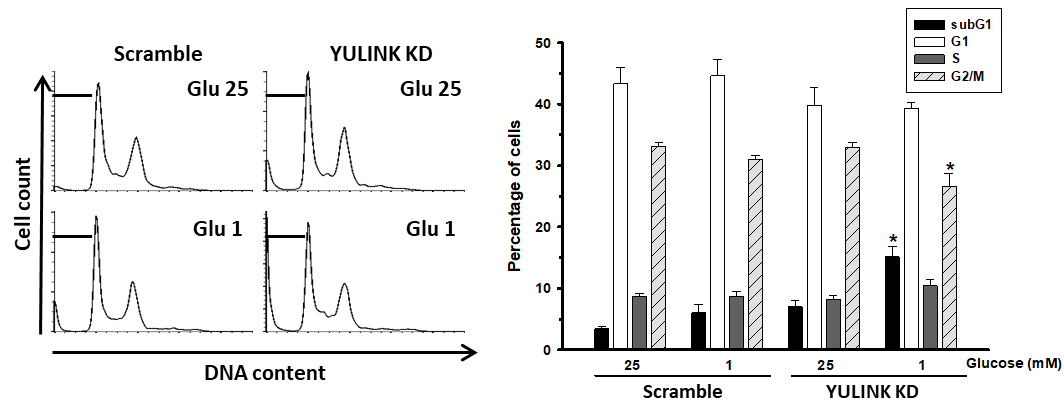
**

**YULINK suppression enhanced glucose restriction-triggered cell death in HA22T cells.** HA22T cells were cultured in Minimum Essential Medium (MEM) supplemented with 10% fetal bovine serum (FBS), 2 mM L-glutamine, 100 U/mL penicillin, and 100 μg/mL streptomycin. Cells were maintained in a humidified incubator at 37 °C and 5% CO₂. HA22T cells with or without YULINK knockdown were maintained under high glucose (25 mM) or glucose restriction (1 mM) conditions for 12 h before harvesting. The cell cycle distribution was analyzed by flow cytometry. In each panel, cell cycle profiles are presented together with bar graphs, indicating the cell distribution in each phase of the cell cycle. Values in the bar graphs represent the mean of three independent experiments ± S.D. *P<0.05 indicates significant differences between glucose 25 mM and 1 mM within the Scramble and YULINK KD group.

**Supplementary Figure S3**

**
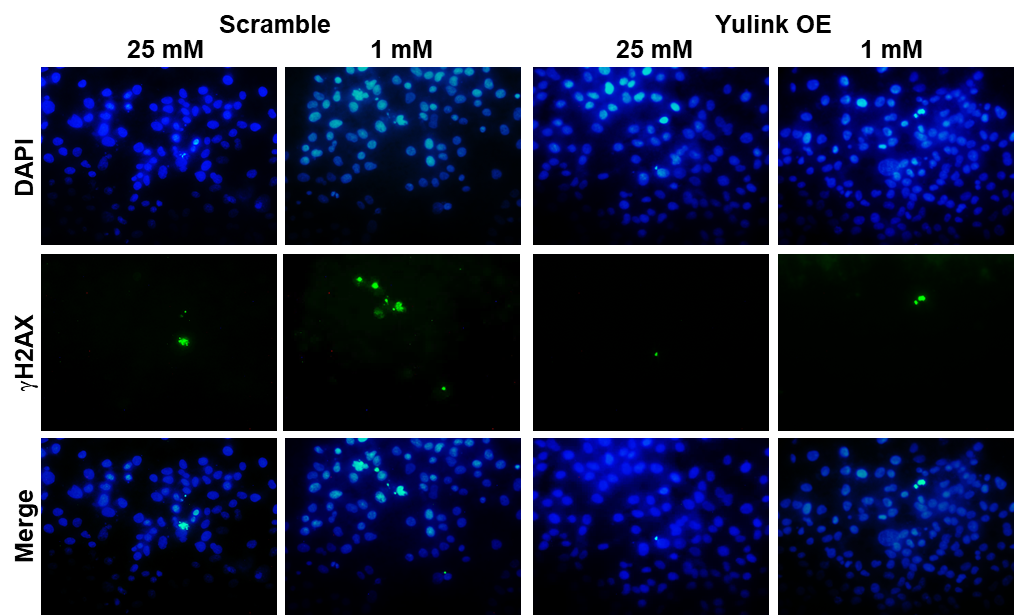
**

**Overexpression of YULINK reduced γ-H2AX expression in Huh7 cells under glucose restriction.** Huh7 cells with or without YULINK overexpression were maintained under 25 mM glucose or glucose restriction (1 mM) for 24 h before fixing and staining for DAPI (blue signal) and phosphorylated pSer139 H2AX (γ-H2AX, green signal).

**Supplementary Figure S4A**

**
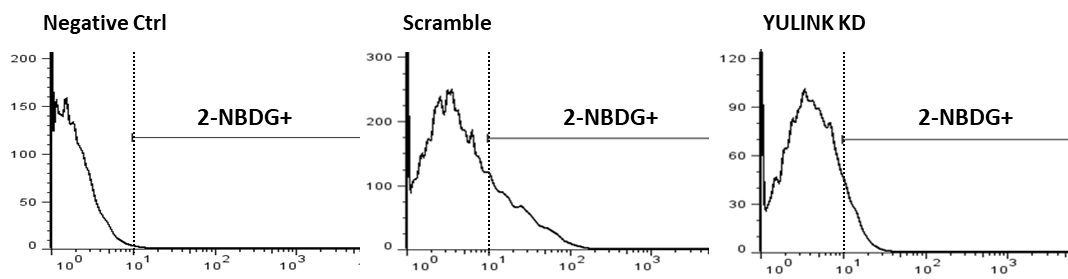
**

**YULINK suppression disturbed glucose uptake in HA22T hepatocellular carcinomas.** HA22T cells with or without YULINK knockdown were treated with 2-NBDG for 60 min and subjected to flow cytometric analysis.

**Supplementary Figure S4B**


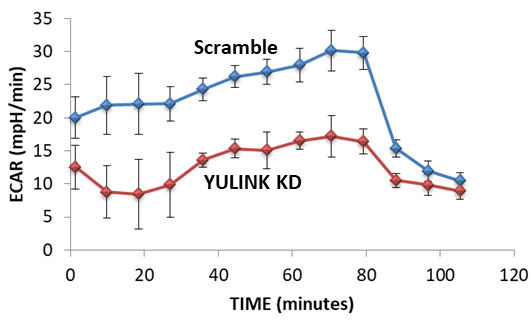


**YULINK suppression disturbed functional glycolysis in HA22T hepatocellular carcinomas.** Extracellular acidification rate of HA22T cells with or without YULINK knockdown was examined using a Seahorse Bioscience XF24 Extracellular Flux Analyzer and a Seahorse XF Glycolysis Stress Test Kit.

**Supplementary Figure S5**


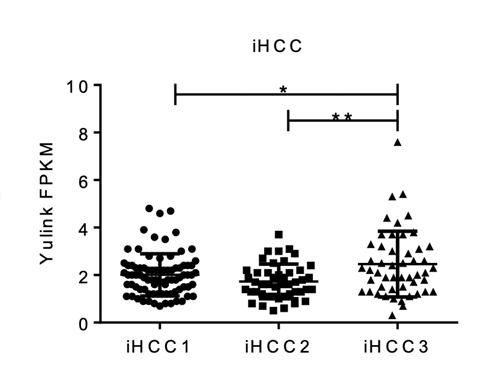


**YULINK expression in different iHCC type.** One-way ANOVA analysis showing YULINK expression level (FPKM value) with respect to TCGA LIHC data. YULINK expression level of 179 HCC patients was categorized as iHCC (iHCC 1, iHCC2 and iHCC3) subtypes [36]. Data were statistically analyzed by ANOVA , *: P<0.05, **: P<0.01.
